# Development and Validation of a Prognostic Model for Multi-Drug-Resistant Non-Hospital-Acquired Bloodstream Infection

**DOI:** 10.3390/antibiotics12060955

**Published:** 2023-05-24

**Authors:** Emanuele Pivetta, Silvia Corcione, Paolo Peasso, Irene Cara, Alberto Capodanno, Andrea Brussino, Paolo Petitti, Eleonora Galli, Maddalena Galmozzi, Valeria Ghisetti, Rossana Cavallo, Franco Aprà, Enrico Lupia, Francesco Giuseppe De Rosa, Giuseppe Montrucchio

**Affiliations:** 1 Department of Medical Sciences, Division of Emergency Medicine and High Dependency Unit, University of Turin, 10126 Turin, Italy; enrico.lupia@unito.it; 2 Department of Medical Sciences, Infectious Diseases, University of Turin, 10126 Turin, Italy; silvia.corcione@unito.it (S.C.); francescogiuseppe.derosa@unito.it (F.G.D.R.); 3 Department of General and Specialized Medicine, Internal Medicine 2, Città Della Salute e Della Scienza di Torino University Hospital, 10126 Turin, Italy; paolopeasso@gmail.com; 4 Residency Program in Emergency Medicine, University of Turin, 10126 Turin, Italy; 5 Internal Medicine Unit, San Giovanni Bosco Hospital, Local Healthcare Unit of the Città di Torino, 10154 Turin, Italy; albertocapodanno@gmail.com; 6 Internal Medicine, Ordine Mauriziano Hospital, 10128 Turin, Italy; andrea.brussino@gmail.com; 7 Emergency Department, Maria Vittoria Hospital, Local Healthcare Unit of the Città di Torino, 10144 Turin, Italy; paolo.petitti@gmail.com; 8 Residency Program in Internal Medicine, University of Turin, 10126 Turin, Italy; eleonora.galli@unito.it; 9 Emergency Department, Ordine Mauriziano Hospital, 10128 Turin, Italy; maddalena.galmozzi@gmail.com; 10 Molecular Biology and Microbiology Unit, Amedeo di Savoia Hospital, Local Healthcare Unit of the Città di Torino, 10149 Turin, Italy; valeria.ghisetti@unito.it; 11 Microbiology and Virology Unit, Città Della Salute e Della Scienza di Torino University Hospital, 10126 Turin, Italy; rossana.cavallo@unito.it; 12 High Dependency Unit, Emergency Department, San Giovanni Bosco Hospital, Local Healthcare Unit of the Città di Torino, 10154 Turin, Italy; franco.apra@aslcittaditorino.it; 13 Department of Medical Sciences, Internal Medicine 2, University of Turin, 10126 Turin, Italy; giuseppe.montrucchio@unito.it

**Keywords:** bloodstream infection, multidrug resistance, healthcare associated bacteremia, prognostic models

## Abstract

Bloodstream infections (BSI) are an increasing cause of admissions to hospitals. Non-hospital-acquired BSI are defined by blood cultures that are positive less than 48 hours after admission, but a relevant difference exists between community-acquired and healthcare-associated (HCA) BSI in terms of risk of multidrug resistance (MDR). We planned a retrospective study in three different cohorts in order to develop and to temporally and spatially validate an easy and rapid prognostic model for identifying MDR non-hospital-acquired (non-HA) BSI. The pathogens most involved in BSI are *Staphylococcus* spp. and *Escherichia coli*, responsible for about 75% of all MDR isolated. The model includes age, gender, long-term care facility admission, immunocompromise, any recent invasive procedures and central line placement, recent intravenous treatment and antibiotic treatment. It shows an acceptable performance, especially for intermediate probabilities of MDR infection, with a C-index of 70%. The model was proposed in a nomogram that could allow better targeting of antibiotic therapy for non-HA BSI admitted in hospital. However, it should be further validated to determine its applicability in other populations.

## 1. Introduction

Considering population-based studies conducted in North America and Europe between 2000 and 2015, the bacteriemia or bloodstream infection (BSI) incidence ranges between 113 and 220 per 100,000 persons. However, these studies do not include BSI due to difficult-to-culture microorganism such as *Borrelia*, *Bartonella*, *Coxiella*, *Rickettsia* and *Mycobacteria*; the addition of these pathogens may produce a more realistic estimate of BSI incidence and burden, in particular regarding community-onset disease [1]. 

Key pathogens in studies from high-income countries are *S. aureus*, *Escherichia coli*, *Klebsiella* spp., *Pseudomonas aeruginosa*, *Enterococci*, *Streptococci* and *Coagulase-negative staphylococci*. The spectrum of organisms is different for community-acquired (*Streptococcus pneumoniae*, other *Streptococci* and *Escherichia coli*) versus healthcare-associated infections (*Pseudomonas aeruginosa* and *Staphylococci*) [1]. Furthermore, it is important to keep in mind the worldwide increase in the proportion of antimicrobial resistance among the pathogens causing bloodstream infections [2]. According to surveillance data from 2016 reported by WHO/EARS-Net, the European countries with a carbapenem resistance rate ≥ 25% among *Klebsiella pneumoniae* blood and central nervous system isolates include Italy, Greece, Serbia, Romania, Belarus and Turkey. The situation outside Europe is highly heterogeneous [1].

BSI results in significant short-term morbidity and mortality worldwide, but is also associated with poor long-term outcome when compared to matched controls [3,4].

### 1.1. Pathogen-Related Prognostic Risk Factors

Prognosis partly varies according to the pathogen. A recent systematic review established that the most important predictors of mortality in patients with MRSA (*Methicillin-Resistant Staphylococcus aureus*) bloodstream infection are age, patient condition, timing and appropriateness of antimicrobial treatment, surgical intervention and disease severity as evaluated by the APACHE II score [5]. The incidence of BSI due to *Escherichia coli* is higher than that of *Staphylococcus aureus* bacteremia, but the mortality is lower: the in-hospital and 30-day case-fatality is approximately 10–15%; mortality is highly dependent on age, hospital acquisition and co-morbidity, initial presentation with septic shock/severe disease and resistance to fluoroquinolones and third-generation cephalosporin resistance [1]. Although less frequent than E. coli among bacteremia isolates, BSI due to extended-spectrum beta-lactamase (ESBL)-positive *K. pneumoniae* carries a worse prognosis, including more frequent intensive care unit admission and higher 30-day case-fatality or in-hospital mortality. Many European and North American studies show an association between ESBL-positive *Enterobacterales* and both excess length of stay and increased mortality rate [1,6,7,8]. *Klebsiella pneumoniae* is also the most prominent among carbapenem-resistant Gram-negative bacteria causing BSI. The case-fatality of BSI due to carbapenem-resistant *Klebsiella* is higher than that due to carbapenem-susceptible *K. pneumoniae*, and the likelihood of initially inappropriate therapy and of suboptimal definitive therapy was significant and a major prognostic factor for poor outcomes before the availability of newer antibiotics. A particularly high risk of fatal outcome after BSI due to carbapenem-resistant Gram-negative bacteria in general has been reported for immunocompromised patients [1].

### 1.2. Host-Related Prognostic Risk Factors

In patients with a history of malignancy, the most common predictors of mortality among BSI caused by *Enterobacteriaceae* are septic shock, pneumonia and ICU admission; furthermore, children and hematological malignancy are associated with higher mortality [6,7,8]. The relationship between some malignancies and prolonged bacterial infection has already been shown. For example, the etiology of gastric cancer is related to alcohol, smoking and unhealthy diet, but also to *H. pylori* infection and the following changes in gastric microbial population as the cancer develops [9,10,11].

A recent Japanese study shows that an estimated glomerular filtration rate (eGFR) lower than 30 mL/min/1.73 m^2^, procalcitonin of at least 100 ng/mL and primary infectious foci in the gastrointestinal tract or in the respiratory system are independent prognostic factors for short-term survival in patients with BSI [12].

Other studies evaluated prognostic factors in more selected clusters of patients: a machine-learning-based study showed that the most important predictors of death in patients with concomitant candidemia and bacteriaemia are age, serum creatinine level, leukocyte and lymphocyte counts, total bilirubin level, procalcitonin level, endotoxic shock, length of stay in the intensive care unit (ICU), length of stay in the hospital and total parental nutrition [13]; another recent Chinese cohort study indicates that ICU admission, coronary heart disease, biliary infection and the use of tigecycline are independent prognostic factors of 90-day mortality in elderly people (≥65 years old) with both typical (e.g., transplant recipient, hematological malignancy) and atypical (e.g., diabetes mellitus, liver cirrhosis, burns, postoperative) immunosuppression status, and that a decrease in body mass index is a protective factor [14].

### 1.3. Bloodstream Infection Classification

BSI are usually categorized as community-acquired (CA-BSI) or hospital-acquired (HA-BSI) based on the timing of the positivity of blood cultures (i.e., before or after 48 h since the arrival in hospital, respectively) [15]. HA-BSI pose a higher risk of morbidity and mortality compared with CA-BSI. However, an additional, intermediate, category of BSI, healthcare-associated (HCA-BSI), has been proposed [16]. This category is similar to CA-BSI in terms of the timing of culture results, but it also has at least one risk factor from among the following: recent hospitalization (i.e., at least 2 days in the last 3 months); admission in a long-term care facility in the last 30 days; antibiotic treatment (at least 5 days in the last month); intravenous therapy for chronic ulcers in the last month; hemodialysis in the last 30 days; and immunocompromise [15]. The risk of multidrug resistance (MDR) in the non-HA BSI, specifically in the HCA-BSI, has been increasing in the past few years [17].

Due to this possible higher risk of MDR among some non-HA BSI, and specifically among HCA-BSI compared with CA-BSI, we hypothesized the need of a rapid, empiric antibiotic treatment for these patients, and we aimed to develop a prognostic model for identifying non-HA BSI at high risk of MDR pathogens and to internally and externally validate such a model. 

## 2. Results

### 2.1. Demographic Characteristics and Risk Factors

The development cohort included 556 patients enrolled between January 2012 and December 2013. The CA-BSI cases numbered 182 (32.7%), and, based on the blood cultures results, 28 were MDR infections (14.7% of all MDR infections included in the development cohort). The HCA-BSI cases numbered 374 (67.3%), and 162 (85.3% of all MDR infections) were MDR. Female patients numbered 82 and 155 among the CA- and HCA-BSI, respectively (*p* = 0.419), and the median ages were 75 years (range 25–99) and 69 years (range 26–99), respectively (*p* = 0.1). 

The validation cohorts enrolled 609 and 253 patients between January 2014 and December 2015 and between January 2014 and December 2014, respectively, for temporal and spatial external validations (i.e., in the Città della Salute e della Scienza di Torino University Hospital and in the San Giovanni Bosco University affiliated hospital (Local Healthcare Unit of the Città di Torino, Turin, Italy). 

In the cohort used for temporal external validation, the female patients numbered 149 and 121 among the CA- and HCA-BSI cases (*p* = 0.03). The median ages of the enrolled patients were 73.5 years (range 18–97) for CA-BSI and 73 years (range 24–94) for HCA-BSI (*p* = 0.98).

In the spatial external validation cohort, the female patients numbered 65 and 56 among the CA- and HCA-BSI cases (*p* = 0.818). The median ages of the enrolled patients for spatial external validation were 72.4 years (range 18–95) for CA-BSI and 73.4 years (range 25–90) for HCA-BSI (*p* = 0.629).

Table 1 summarizes the baseline characteristics in each cohort, including the risk factors for HCA-BSI.

In the model development cohort, immunocompromise was due to prolonged steroid treatment in 116 patients (20.9%), chemotherapy in 80 (14.4%), and/or radiation therapy in 10 (1.8%) in the last month, and/or treatment for previous transplantation (solid or hematological) or for other medical conditions in 58 patients (10.4%), splenectomy in 11 (2%) and known human immunodeficiency virus infection in 4 (0.7%).

In the temporal and spatial validation cohorts, respectively, prolonged steroid treatment was reported in 66 and 30 patients (10.8% and 11.9%), chemotherapy in 36 and 19 (5.9% and 7.5%), and/or radiation therapy in 3 and 2 (0.5% and 0.8%) in the last 30 days, and/or treatment for previous solid or hematological transplantation or for other conditions in 23 and 11 (3.8% and 4.4%), splenectomy in 3 and 2 (0.5% and 0.8%) and known human immunodeficiency virus infection in 6 and 1 (1% and 0.4%).

Table 2 reports the additional risk factors evaluated for MDR.

Gram-negative agents were detected in 51.6% of the patients in the development cohort, and in 53.7% and 59% of the patients in the temporal and spatial validation cohorts, respectively. 

Table 3 shows the results of MDR pathogens identified in the blood cultures as single agents. A polymicrobial positivity was found in 30, 34 and 21 patients, in the development, temporal and spatial validation cohorts, respectively. 

In the development cohort, 51 patients suffered from septic shock (9.2%), compared with 58 (9.5%) in the temporal validation cohort, and 18 (7.1%) in the spatial validation cohort. 

Mortality risk was 7.7% among CA-BSI and 15% among HCA-BSI in the development cohort (*p* = 0.009), 11.3% and 20.1% (*p* = 0.008), respectively, in the temporal validation cohort, and 10.4% and 16.8% (*p* = 0.139), respectively, in the spatial validation cohort. 

### 2.2. Prognostic Model Development

Based on the data available in the development cohort and previous data already published and suggested as potential predictors in the international literature, we identified eight predictors of MDR among non-HA BSI: age, gender, long-term care facility admission, immunocompromise, any recent invasive procedures, any central venous catheterization, recent intravenous treatment and antibiotic treatment. This model showed an area under the receiver operating characteristic curve of 71.6% (95% confidence interval 61.8–72.4%). 

Table 4 shows the odds ratios (OR) of MDR for each of these variables in the uni- and multivariable models. 

**Figure 1:**
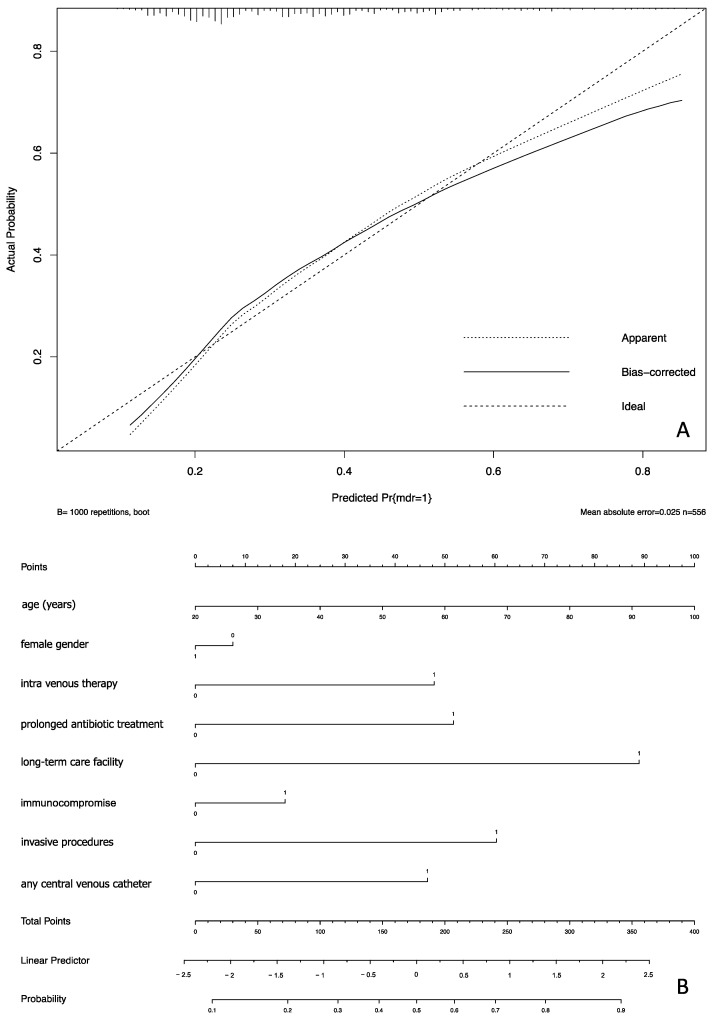
Calibration (**panel A**) of the prognostic model and its nomogram (**panel B**) including the eight selected covariates.

Figure 1 shows the calibration of the developed prognostic model along with its nomogram. It was built using the already reported covariates. Such a model had a C-index of 70%.

### 2.3. Model External Validation

#### 2.3.1. Temporal Study

The prognostic model built in the development cohort was validated in two different cohorts. One of them was collected in the same hospital (i.e., Città della Salute e della Scienza University Hospital, Molinette) but over a different time period. This cohort included 609 patients with 177 MDR BSI.

**Figure 2:**
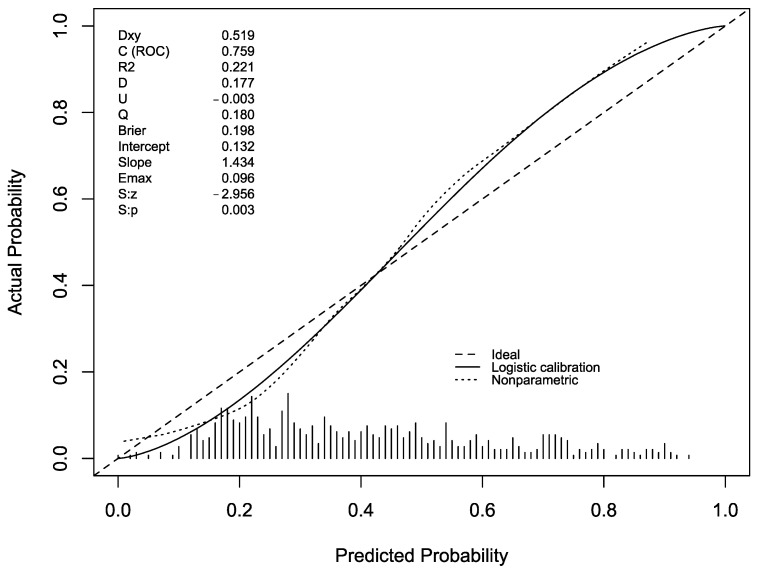
Model temporal external validation, predicted versus observed probability of MDR BSI plot.

Figure 2 reports the predicted versus observed probability of MDR BSI (calibration) as calculated in this temporally different cohort. 

The C-index was 75.9%.

#### 2.3.2. Spatial Study

A third cohort was collected for performing an additional external validation of the proposed prognostic model. This cohort referred to an entire single year of blood cultures including 253 patients and 83 MDR BSI infections. The data were collected in a different University-affiliated hospital in Turin, Italy (San Giovanni Bosco Hospital—Local Healthcare Unit of the Città di Torino). 

**Figure 3:**
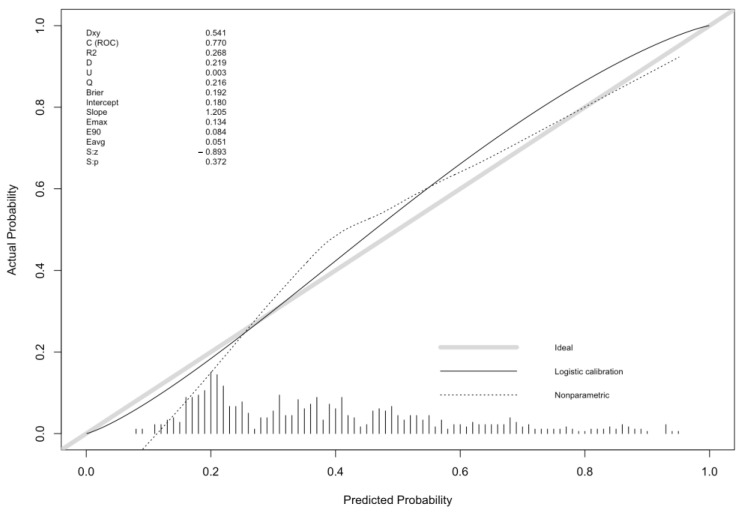
Model spatial external validation, predicted versus observed probability of MDR BSI plot.

Figure 3 shows the calibration of the model in such a cohort (spatially external validation). The C-index was 77%.

## 3. Discussion

We have developed and validated an eight-variable prognostic model for the risk of MDR non-HA BSI in three different cohorts of patients admitted to hospital. 

Our results suggest this possible prognostic model for high risk for MDR BSI among the non-HA BSI. This model was developed and then separately validated in three different cohorts. The external validation was repeated using two sets of data; one of them was collected in the same University Hospital, the second one in a different University-affiliated hospital in Turin, Italy. The double validation was performed in order to better understand the validity of the prognostic model. 

This model was summarized in a nomogram, a fast-performing tool for assessing the risk of MDR at a patient’s admission to hospital. 

The need to identify high risk of MDR among non-HA BSI, usually due to frequent contacts with the healthcare systems (for this reason, called HCA-BSI), is linked to the different MDR risk within the large group of patients presenting for BSI and with a blood culture positivity before 48 h after admission. During the past few decades, the number of patients who were treated and followed up outside the hospital has gradually increased, and this has changed the number of patients affected by BSI and the most frequent pathogens [1,2]. For these reasons, the idea of “healthcare-related” infection is a relatively new one, but it is crucial for the correct management of patients affected by BSI [15].

Our data confirmed a significant different mortality risk for CA- and HCA-BSI, except for the spatial validation cohort, where the difference was present but without any significance; likely this is due to its smaller size compared with the other 2 cohorts. 

Moreover, as previously reported in the international literature, the pathogens most likely involved in BSI are *Staphylococcus* spp., and *Escherichia coli* [19]. In our study, they are responsible of about 75% of all MDR isolated.

The presented model was developed using readily available data. Unlike previous studies and prognostic models [12,13,14,20,21], all parameters reflect patient demographics (i.e., age and gender) and recent medical history data. It shares some parameters with the healthcare-associated infection definition [15] by adding information about the need of central lines and any recent invasive procedures. Some of these procedures might be very frequent, as the placement of a urinary catheter, not only in the Emergency Department for acute illness, but also among out-of-hospital patients for worsening of chronic diseases (e.g., solid or hematologic neoplasm). 

In general, the prognostic model shows a good performance across the three validation cohorts. In the development cohort, it had a better performance for the intermediate risk of MDR infection, between 20 and 70%. In the validation cohorts, performance remained acceptable for the same probabilities of MDR infection.

Our study has limitations. Firstly, we collected data in two large hospitals, a University one and a University-affiliated one. The patients referred to similar hospitals might be partially selected by some types of diseases (e.g., hematological and/or advanced oncological diseases) that are not usually treated in all hospitals. This situation might then have an impact on the generalizability of our model as well as the absence of comorbidities in the prognostic models. The inclusion of these comorbidities might have an impact on model performance.

Secondly, we restricted our analysis to the first blood cultures collected for each patient included in the study (for each cohort). It is likely that some patients might be admitted in hospital more than once during each study period, specifically for patients suffering from chronic diseases with high risk of immunocompromise. However, we chose to externally validate our model twice, and its performance remained acceptable. Based on the features of the cohorts we used, the model is not applicable to different settings like out-of-hospital clinics or intensive care units. 

The study also presents some strengths. It is based on three large cohorts collected in different period of time. This allowed us to develop the model with enough degrees of freedom and to externally validate it. This second procedure was performed twice. The first external validation was based on data collected in the same hospital where the development cohort was collected, but in a different time frame (2012–2013 vs. 2014–2015). A temporal validation, performed in more-recently treated patients, is a possible way for assessing model performance [22]. However, for better understanding the real performance of the suggested prognostic model, we chose a second external validation, a spatial (or, so called, geographical) one, in a different site. This second process allowed us to confirm our results, but, as already well described in the literature [22], with the drawback of a smaller cohort than the development and temporal validation cohorts. This also allowed us to underline the general consistency of the model. 

## 4. Materials and Methods

The present study was held in the Città della Salute e della Scienza di Torino University Hospital and in the San Giovanni Bosco University affiliated hospital (Local Healthcare Unit of the Città di Torino), Turin, Italy. Data were retrospectively collected based on the laboratory information system results on positive blood cultures.

The hospital Ethics Committee approved the study protocol (n. 0033874). Informed consent was waived because of the retrospective nature of the study and because the analysis used anonymous data. All data were collected from the laboratory information system, and then from medical records, by five investigators (A.C., A.B., P. Pet., E.G. and M.G.).

The primary outcome of the study was to predict the risk of MDR infection among the non-HA BSI (i.e., CA- and HCA-BSI).

For the development cohort, we recruited adult patients (i.e., age ≥ 18 years) with positive blood cultures admitted at the Città della Salute e della Scienza di Torino, Molinette Hospital between January 2012 and December 2013. Samples collected in intensive care, neurosurgery and cardiac surgery units were excluded. Patients with multiple admissions were included in the study only at their first admission, and data relative to subsequent admissions were excluded. 

Since the study outcome was MDR in non-HA BSI, we excluded all positive cultures collected more than 48 hours after admission to hospital, as well as those collected in the Emergency Department while patients were waiting for admission due to hospital overcrowding.

Demographic and clinical characteristics of enrolled patients were reported as median and range for continuous data or number and percentage for ordinal data, as appropriate. 

The continuous and the categorical variables were tested by using the non-parametric Wilcoxon rank-sum test for unmatched samples, and the Chi-squared or Fisher’s exact test, as appropriate, respectively [23,24].

Multivariable analysis was used to predict MDR among non-HA BSI in the development cohort. Covariates tested were age, gender, long-term care facility admission, immunocompromise, any recent invasive procedures, any central venous catheterization, recent intravenous treatment and antibiotic treatment. The decision to include these variables was taken a priori, based on prior knowledge and the available literature [15,16,22,25,26,27]. The number of predictors was kept to a minimum in order to avoid overfitting and to maximize the use of the predictive model in future applications. 

The accuracy of the proposed model was reported as the area under the receiver operating characteristic curve (AUC ROC) [28]. The model was internally validated by bootstrapping (1000 replications) to obtain overfit-corrected estimates of discrimination and calibration [22].

The discrimination ability was measured using the optimism-corrected C-index [29]. Apparent and internally validated (after a 1000 replication bootstrapping procedure) calibration was visually assessed by evaluating the plot of the observed vs. predictive probability of MDR BSI [22].

A second cohort, using the same inclusion criteria, was identified in the same hospital (Città della Salute e della Scienza di Torino, Molinette Hospital), between January 2014 and December 2015. This cohort was used for an external validation of the model in a different time frame (i.e., temporal validation).

A third cohort, again based on the same criteria for enrollment, was collected using the data referring to a different University-affiliated hospital, the San Giovanni Bosco Hospital, Local Healthcare Unit of Città di Torino, Turin, Italy. This hospital shares a similar population of patients and the period of collection was the same as that for the temporal external validation cohort in the Città della Salute e della Scienza di Torino, Molinette Hospital. This last cohort was also used for external validation (i.e., between January 2014 and December 2015, spatial external validation of the model). 

**Figure 4:**
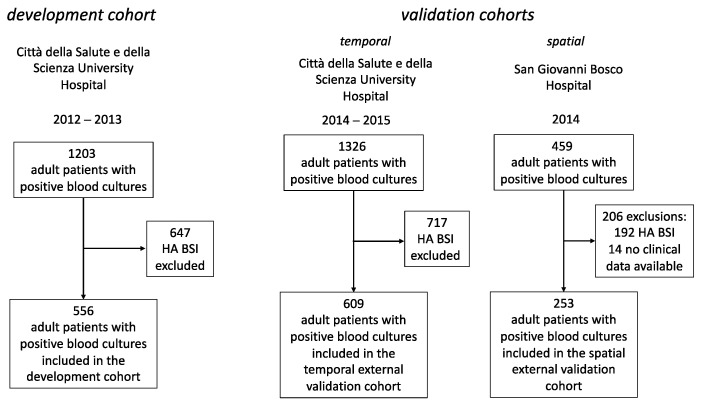
Diagram illustrating the collection of data for the three used cohorts (HA BSI, hospital acquired bloodstream infection).

Figure 4 briefly summarizes the data collection for these three cohorts. 

A nomogram, a graphical presentation, was proposed as an easy-to-use tool for applying the prognostic model. It is based on the calculation of the linear predictor. A reference line allows the user to score each variable for the patient and identify a total score corresponding to the linear predictor that has to be transformed in the prediction read at the bottom of the nomogram [30,31].

Antimicrobial susceptibility was determined by a commercially available microdilution assay (Panel NMDR, MicroScan^®^ WalkAway^®^ 96 Plus; Nyon, Beckman Coulter, Switzerland), and ceftazidime/avibactam minimum inhibitory concentrations (MICs) were confirmed by Etest (bioMérieux, Paris, France). Susceptibility data were interpreted according to current European Committee on Antimicrobial Susceptibility Testing (EUCAST) breakpoints [32].

Analyses were performed using STATA 17 (Stata Corp TX, College Station, TX, USA) and R version 3.6.3 (The R Foundation for Statistical Computing, 2020).

## 5. Conclusions

This study proposed the development and double validation of a prognostic model for MDR non-HA BSI in hospital admitted adult patients. This model, along with the easy-to-perform nomogram, could allow better targeting of empiric antibiotic therapy for BSI before the results of blood cultures become available. However, further prospective studies are needed to confirm the present results and to better define the impact of each covariate.

## Figures and Tables

**Table 1:** Baseline characteristics of patients enrolled in each cohort and risk factors for HCA-BSI.

	Development Cohort (N = 556)	Temporal External Validation Cohort (N = 609)	Spatial External Validation Cohort (N = 253)
Median age, years (IQR) ^1^	70.5 (19)	73 (18)	72.7 (20.1)
Male/female ratio	1.35	1.26	1.1
Mortality in hospital ^2^	69 (12.4%)	75 (15.7%)	34 (13.4%)
Long-term care facility	31 (5.6%)	51 (8.4%)	22 (8.7%)
Recent hospitalization	238 (42.8%)	148 (24.3%)	61 (24.1%)
Recent antibiotic treatment	113 (20.3%)	123 (20.2%)	48 (19%)
Recent intravenous therapy	117 (20%)	120 (19.7%)	40 (15.8%)
Hemodialysis	17 (3.6%)	15 (2.5%)	5 (2%)
Immunocompromise ^3^	146 (26.3%)	107 (17.6%)	46 (18.2%)
Diabetes	134 (24.1%)	175 (28.7%)	66 (26.1%)
COPD ^4^	63 (11.3%)	79 (13%)	33 (13%)
Any type of cardiopathy	172 (30.9%)	203 (33.3%)	85 (33.6%)
Chronic renal failure	118 (21.2%)	102 (16.8%)	40 (15.9%)
Liver cirrhosis	48 (8.6%)	35 (5.8%)	26 (10.3%)
Cerebrovascular disease	98 (17.6%)	88 (14.5%)	42 (16.6%)
Any type of solid tumor	93 (16.7%)	95 (15.6%)	34 (13.8%)
Any type of hematological tumor	45 (8.2%)	34 (5.6%)	18 (7.1%)
Neutropenia ^5^ at arrival in hospital	33 (5.9%)	12 (2%)	8 (3.2%)

^1^ IQR, interquartile range; ^2^ in-hospital mortality during the index admission; ^3^ any type of immunocompromised state, such as recent (i.e., in the last 30 days) or ongoing chemotherapy and/or radiotherapy and/or immunosuppressive therapy; prior solid organ or bone marrow transplant, splenectomy, congenital or acquired immunodeficiency; ^4^ chronic obstructive pulmonary disease (COPD); ^5^ neutropenia, defined as an absolute neutrophil count of less than 0.5 × 10^9^/L [18].

**Table 2:** Additional risk factors for MDR evaluated for the prognostic model.

	Development Cohort (N = 556)	Temporal External Validation Cohort (N = 609)	Spatial External Validation Cohort (N = 253)
Any type or recent surgical operation ^1^	45 (8.1%)	57 (9.4%)	24 (9.5%)
Any valvular prosthesis	24 (4.3%)	49 (8%)	39 (15.4%)
Any vascular prosthesis	8 (1.4%)	22 (3.6%)	7 (2.8%)
Any central venous catheterization ^2^	154 (27.7%)	71 (11.7%)	52 (20.6%)
Any invasive procedures ^3^	124 (22.3%)	63 (10.34%)	40 (15.8%)

^1^ any type of surgical operations in the last 3 months; ^2^ central lines including those needed for urgent hemodialysis; ^3^ any invasive procedures in the last 4 days, including urinary catheter, any type of drainage, percutaneous endoscopic gastrostomy, enteral and/or endovascular nutrition or arterial catheterization with or without additional procedures.

**Table 3:** MDR agents isolated in the blood cultures in the three cohorts used for the prognostic model.

	Development Cohort (N = 556)	Temporal External Validation Cohort (N = 609)	Spatial External Validation Cohort (N = 253)
*Staphylococcus aureus*	47 (24.7%)	42 (23.7%)	18 (21.9%)
*Staphylococcus epidermidis*	15 (8.5%)	17 (9.6%)	10 (12.2%)
*Staphylococcus hominis*	24 (12.6%)	25 (14.1%)	12 (14.6%)
*Coagulase-negative Staphylococci*	6 (3.2%)	6 (3.4%)	3 (3.7%)
*Enterococcus faecium*	8 (4.2%)	3 (1.7%)	2 (2.4%)
*Escherichia coli*	61 (32.1%)	70 (39.5%)	30 (39%)
*Klebsiella pneumoniae*	12 (6.3%)	10 (5.7%)	4 (4.9%)
*Pseudomonas aeruginosa*	4 (2.2%)	4 (2.3%)	1 (1.2%)

**Table 4:** Variables included in the logistic models for multidrug-resistant bloodstream infection risk (age, gender, long-term care facility admission, immunocompromise, any recent invasive procedures, central venous catheterization, intravenous treatment and antibiotic treatment).

	Univariable Model OR	Multivariable Model OR
Age (year)	1.01 (0.99–1.02)	1.02 (1.00–1.03)
Female gender	0.94 (0.66–1.34)	0.9 (0.62–1.34)
Long-term care facility admission	3.8 (1.78–8.1)	3.3 (1.46–7.43)
Immunocompromise ^1^	1.5 (1.05–2.16)	1.27 (0.83–1.94)
Any recent invasive procedures ^2^	2.27 (1.51–3.42)	2.25 (1.45–3.47)
Any central venous catheterization ^3^	2.46 (1.68–3.61)	1.87 (1.18–2.94)
Recent intravenous treatment	3 (1.97–4.56)	1.9 (1.16–3.11)
Recent antibiotic treatment	2.37 (1.56–3.61)	2 (1.26–3.17)

^1^ any type of immunocompromised state, such as recent (i.e., in the last 30 days) or ongoing chemotherapy, and/or radiotherapy, and/or immunosuppressive therapy; prior solid organ or bone marrow transplant, splenectomy, congenital or acquired immunodeficiency. ^2^ any invasive procedures in the last 4 days, including urinary catheterization, any type of drainage, percutaneous endoscopic gastrostomy, enteral and/or endovascular nutrition, arterial catheterization with or without additional procedures. ^3^ central lines including those needed for urgent hemodialysis.

## Data Availability

The data are available upon reasonable request to the corresponding author.
